# Metal-Assisted Chemical Etching of p‑type Si Nanopillars with Doping Level of 10^20^ cm^–3^

**DOI:** 10.1021/acsaelm.5c02158

**Published:** 2025-12-05

**Authors:** Federico Giulio, Luca Calciati, Riccardo Santamaria, Eleonora Bonaventura, Maurizio Acciarri, Emiliano Bonera, Dario Narducci

**Affiliations:** Department of Materials Science, 189823 University of Milano Bicocca, via R. Cozzi 55, I−20125 Milan, Italy

**Keywords:** silicon nanopillars, Metal-Assisted Chemical Etching, nanowires, thermoelectricity, solar cells, sensors

## Abstract

Silicon nanopillars (SiNPs) are quasi-1D Si nanostructures extending normally to the substrate. As Si nanowires, SiNPs display large surface-to-volume ratios and a remarkable reduction of their thermal conductivity by almost a factor one-hundred compared to bulk Si. SiNPs have found applications in energy-related fields, including thermoelectrics and photovoltaics, and in chemical sensing and biosensing. Among the available preparation techniques, Metal-Assisted Chemical Etching (MACE) has been extensively used to prepare high density SiNP forests, as the method is facile and scalable. However, while MACE lets easily obtain low-to-medium doped SiNPs, *p*–type SiNPs with doping level ≥ 10^20^ cm^–3^, essential for energetic applications, could never be obtained. In this paper we provide evidence that this is related to the competition between metal-assisted and noncatalyzed etching. Thus, guided by a model of the silicon–solution and silicon–metal–solution interfaces, we could develop a strategy that enabled for the first time the preparation by MACE of *p*–type SiNPs with doping levels as large as 10^20^ cm^–3^, using Au as the catalyst and Na_2_S_2_O_8_ as the oxidizing agent. The possibility of preparing heavily doped SiNPs largely extends MACE application to critical technological fields spanning from thermoelectrics and photovoltaics to batteries and sensing.

## Graphical abstract

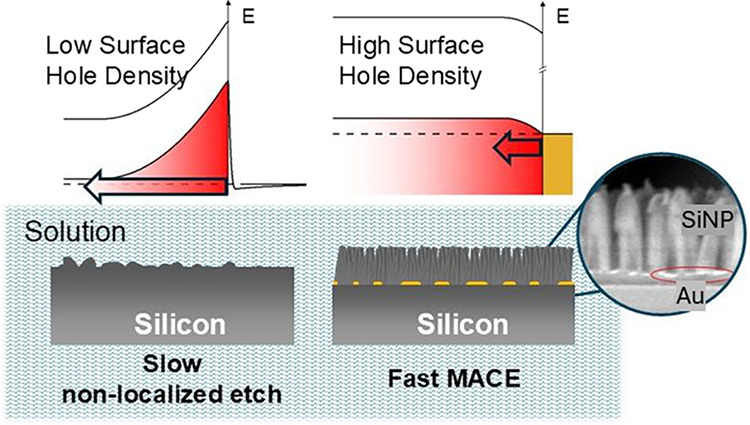

## Introduction

Silicon nanostructures have been the subject of intense research for their applications in many fields. Si nanowires (NWs) attracted the attention for their enhanced thermoelectric figure of merit due to the reduced thermal conductivity.^1,2^ In photovoltaics, Si nanopillars (SiNPs), namely NWs oriented normally to the Si surface, found application as antireflective finishing^3,4^ while they promised enhanced energy storage in batteries.^5,6^ Beyond energy-related applications, SiNPs have been also extensively used in the making of chemical sensors^7^ and biosensors.^8^ In most cases, applications call for heavily doped SiNPs. For thermoelectrics, the optimal doping level for Si is known to be around 10^20^ cm^–3^.^9,10^ In their use in batteries as anodes, efficient charge transport from the current collector to the NWs is necessary for proper battery cycling. Even when acting as finishing of Si solar cells, doping control enables the use of SiNPs at one time as antireflective texture and as cell front contacts.^11^ Of the many techniques available to fabricate SiNPs, Metal-Assisted Chemical Etching (MACE) has raised a remarkable attention because of its simplicity, low cost and scalability.^12^ Born as a method to prepare porous silicon,^13^ first applications of MACE to make SiNPs date 2008.^14,15^ Still, over recent years efforts were made to use it in novel contexts, to achieve unprecedented aspect ratio nanostructures. Although controlling porosity, aggregation and uniformity over large areas remains challenging, Janavicius et al. recently showed how changing MACE parameters (including etchant flow rates, injection and pulse time, chamber pressure and light irradiation) during the etch process enabled the making of complex Si nanostructures of interest for electronic, photonic, quantum, and biomedical devices.^16^ In the same direction but with a different approach, Gupta et al. tailored MACE parameters to fabricate Si nanostructures with applications to energy storage and health care.^17^ Ultralong SiNPs could also be obtained by adapting two-pot MACE.^18^ Alternate solution refresh and water soaking substantially improved the etching rate, leading to the fabrication of SiNPs up to 0.2 mm long successfully deployed in the making of gas sensors. Metal-assisted chemical etching still finds applications in the controlled making of nanoporous silicon^19^ and is used to obtain nanoholes in silcon.^20^ Thus, it is very unfortunate that MACE becomes ineffective when moving from Si doping levels ≤ 2 × 10^19^ cm^–3^ to the 10^20^ cm^–3^ doping range. So far, while standard MACE, using H_2_O_2_/HF solutions, was successful in *p*-type Si doped up to 2 × 10^19^ cm^–3^,^21,22^ only *n*–type SiNPs doped around 10^20^ cm^–3^ could be obtained.^23,24^ Here we report on a MACE process enabling the fabrication of *p*–type SiNPs doped up to 10^20^ cm^–3^. We will show that, counterintuitively enough, MACE prevails over noncatalyzed Si etching when *stronger* oxidants are used. Experiments show that, while using H_2_O_2_ makes metal catalysis ineffective at localizing etching, SiNPs are obtained by using peroxydisulfate with Au as the catalytic metal.

## Results and Discussion

For *n*–type Si, SiNPs were successfully obtained by Wang and collaborators for dopant densities up to 10^20^ cm^–3^ using Ag as catalyst and H_2_O_2_ as oxidant.^24^ As the authors noted, the main problem with heavily doped Si is the competitive etching at the bare Si surface, increasingly narrowing the processing window in terms of reaction time, temperature, and chemical concentrations as well. Figure 1 provides a clue about why Wang’s procedure works properly on *n*–type Si but fails on *p*–type materials. In both cases, Fermi energy in the semiconductor is set either by the metal work function (metal–semiconductor interface) or by the oxidant redox potential (solution-semiconductor interface). Then, in *n*–type Si a potential barrier hinders hole injection both at the bare surface and in the presence of Ag. The opposite happens for *p*–type Si, where bands fold downward at the Ag–Si interface and no barrier opposes either to hole injection *or to their diffusion toward Si bulk*. When no metal is present, instead, the semiconductor–solution interface recalls a *p*-*p* heterojunction, with the space charge being generated by excess holes at the surface. Holes must only overcome a small barrier at the solution side. Then, holes are massively injected into Si, leading to a large hole excess in the accumulation region, whose thickness is about 1 nm. Following Cui and co-workers,^25^ since the etching rate is related to the near-surface hole density, nonlocalized etching is expected to prevail over MACE, in accordance with experiments. Additionally, the use of Ag as catalyst is known to cause secondary nucleation of Ag nanoparticles during the etching process, since metallic Ag is reoxidized by H_2_O_2_. Silver nanoparticles were observed by many scholars to grow also on SiNP walls at any doping level,^12,24,26,27^ causing their supplementary undesired lateral etching. Overall, failure to obtain *p*–type SiNPs is sensibly related to the adverse competition between catalyzed (localized) and noncatalyzed Si etching, which becomes especially critical at very high doping levels.

**Figure 1:**
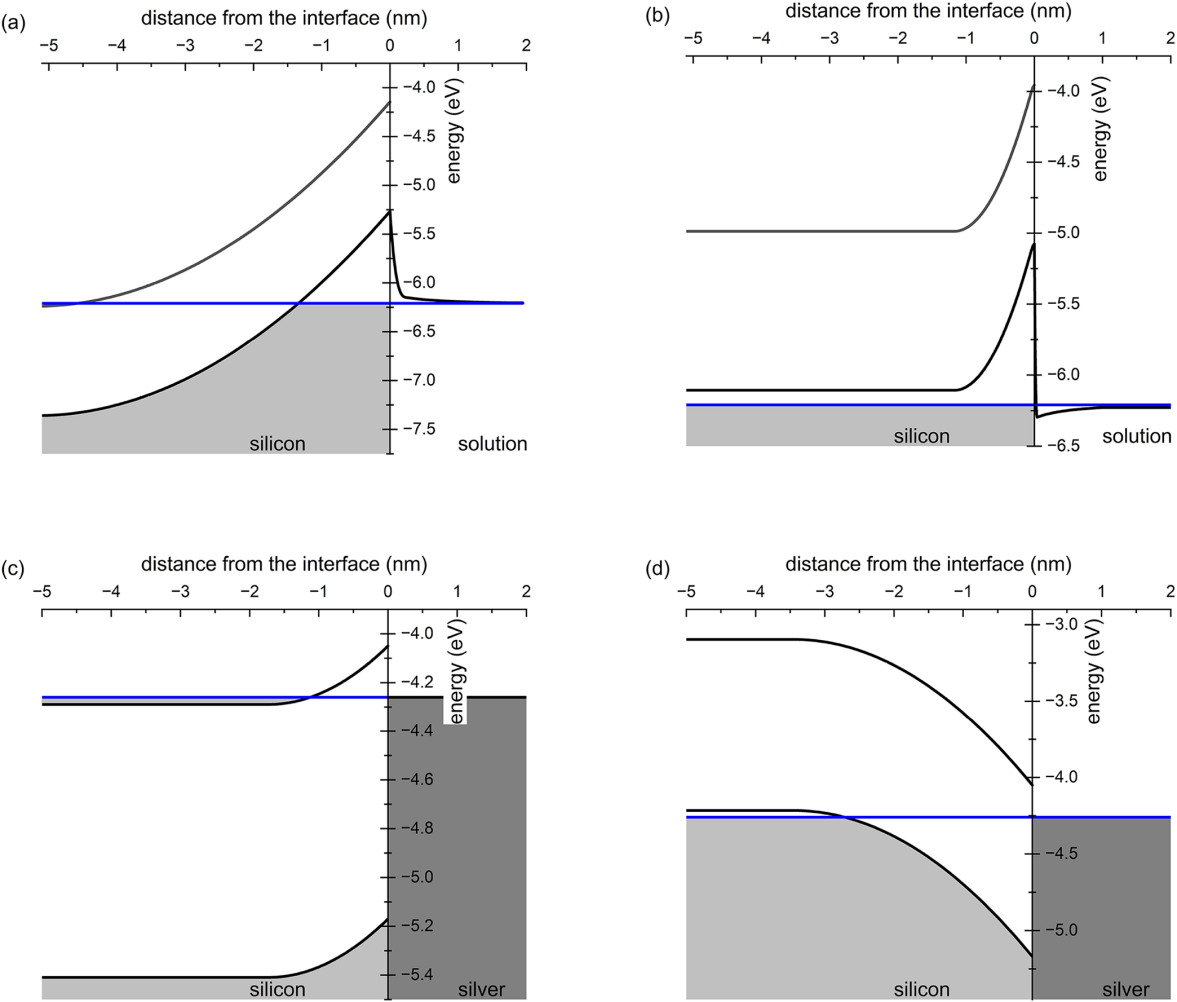
Energy levels across the Si surface for non–catalyzed etching in (a) *n*–type and (b) *p*–type Si; and for Ag–catalyzed etching in (c) *n*–type and (d) *p*–type Si. Dopant density: 10^20^ cm^–3^; oxidizing agent H_2_O_2_, 0.04 M. All energies referred to the vacuum level. The blue lines display the Fermi energy.

To shift etching rates in favor of localized etching, two main handles are available, namely the choice of the catalyst and the redox potential of the oxidizing agent. On the first count, to avoid secondary catalytic metal nucleation, silver may be replaced by a discontinuous gold layer. We note that replacing silver with gold has a negligible impact on the cost-effectiveness of the process, since the needed amount of Au is valued at 0.0025 USD/cm^2^. Concerning instead the oxidant, its choice should aim at decreasing hole density at the direct contact between Si and the electrolytic solution, so to decrease the nonlocalized etching rate.

Figure 2b shows the potential profile in the Si–Au–electrolyte system compared to the Si–electrolyte interface for *increasing* redox potentials Φ_redox_. Remarkably, the width of the barrier in the latter case enlarges for increasing Φ_redox_. Therefore, hole density in the sub–surface region becomes lower when using stronger oxidants. Thus, quite paradoxically but in agreement with experiments, stronger oxidizing agents make the non–MACE etching less competitive, although promoting sub–surface Si oxidation.

**Figure 2:**
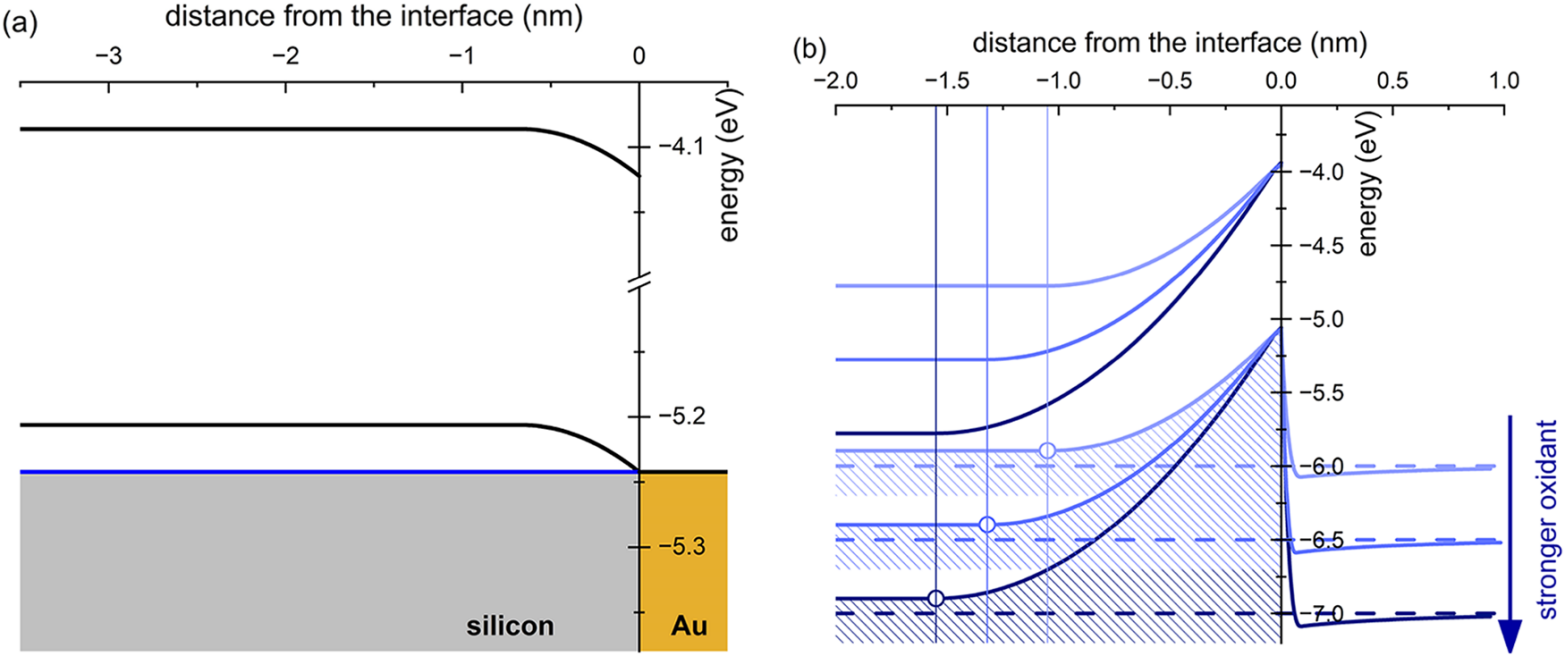
(a) Energy bands at the *p*–type Si–Au interface when hole density is 10^20^ cm^–3^. The blue line displays the Fermi energy; (b) Dependence of band bending at the *p*–type Si–electrolyte interface on redox potential. Note how the accumulation width (open dots) increases with Φ_redox_. All energies referred to the vacuum level.

Cross- and in-plane Scanning Electron Microscopy (SEM) images (Figure 3a,b) confirm the formation of SiNPs on *p*-type Si with a doping level of 10^20^ cm^–3^ (resistivity ≤ 0.001 Ω cm). Gold nanoparticles were detected at the interface between bulk Si and the nanopillars, in accordance to MACE mechanism. The presence of isolated metal particles at SiNP feet was checked not to cause short-circuits.^28,29^ Should they be removed anyway, standard methods including cyanidization to form the soluble Na­[Au­(CN)_2_] salt^30^ or greener processes, replacing cyanide with thiocyanate,^31^ might be considered. SiNPs are strongly agglomerated and, unexpectedly, bundling survives HF etching, which should suppress capillary forces exerting between SiNP oxidized surfaces and the aqueous solution.^32^ SiNP length scales linearly with MACE time, with a formation rate of 23 nm/min (Figure 4). A porous layer also rapidly grows, reaching a steady thickness of 0.7 ± 0.2 μm. Quite notably, also the total etched Si depth, sum of SiNP length and of the porous layer thickness, linearly depends on time, as expected since SiNPs form by localized etching of the porous layer. The formation of the porous layer is consistent with the Si doping level, where the 2-electron etching mechanism prevails,^33^ and in agreement with the very first applications of MACE as an electroless route to prepare porous Si.^13^

**Figure 3:**
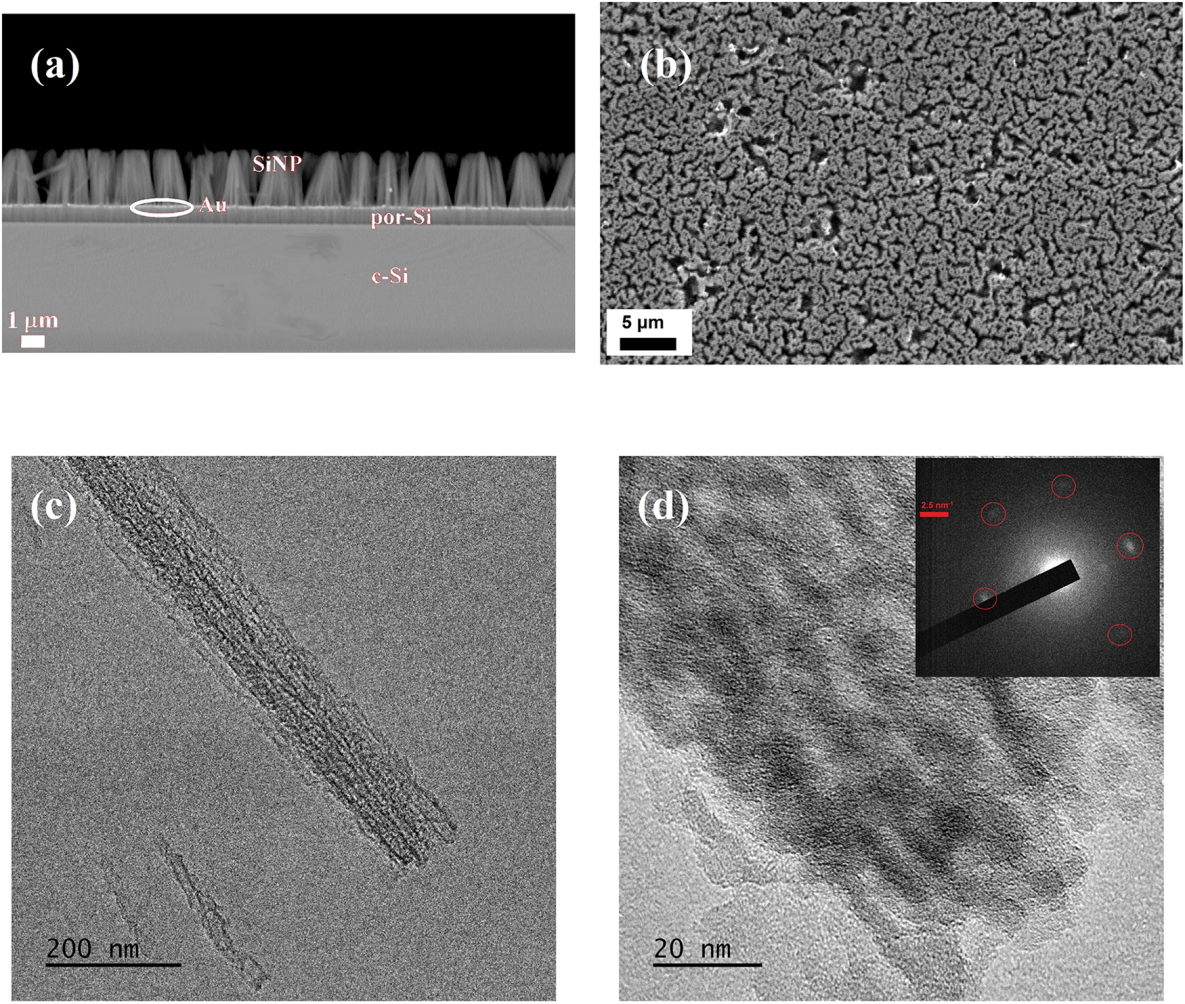
(a) Cross-plane and (b) in-plane micrographs of Si (*p*–type, boron concentration of 10^20^ cm^–3^) after MACE with Na_2_S_2_O_8_, showing *agglomerated* SiNPs. Note Au nanoparticles sitting at the bottom of NWs and the presence of a porous layer at the Si subsurface. (c) Low- and (d) high-resolution TEM image of a nanopillar, showing embedded nanocrystals, as confirmed by the nanodiffractogram (inset).

**Figure 4:**
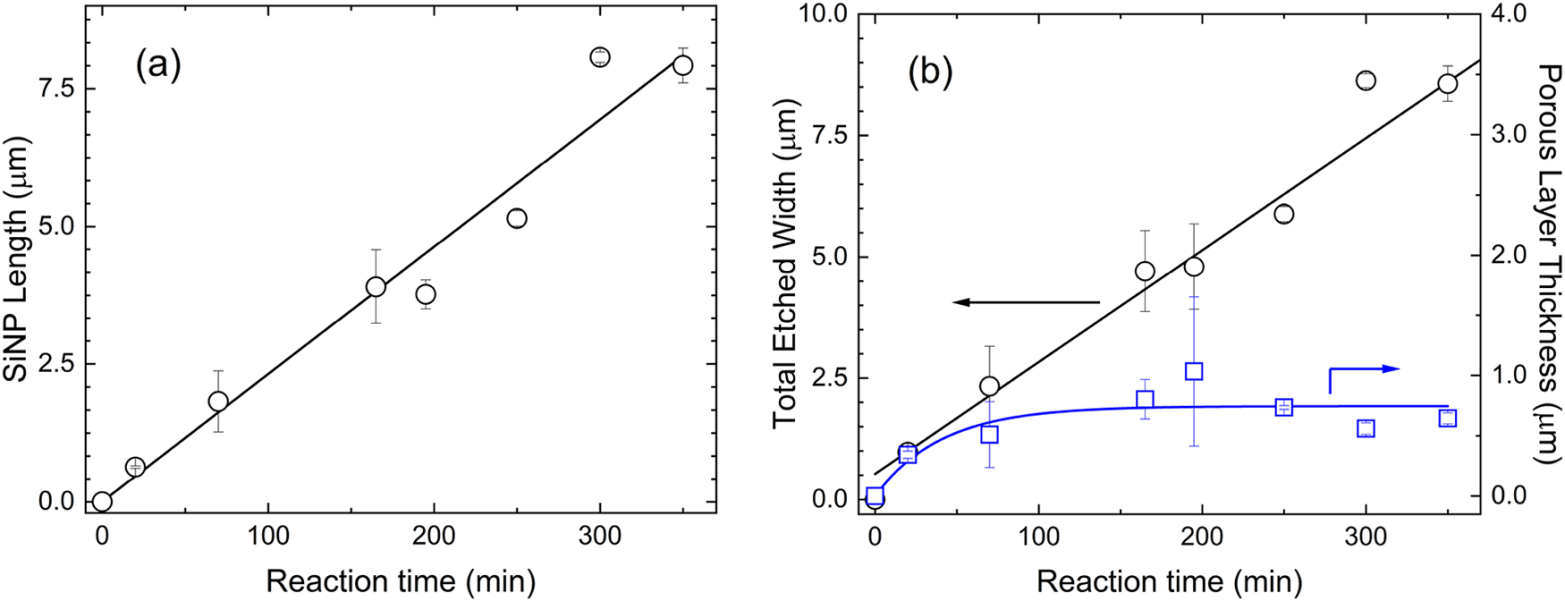
(a) Nanopillar length and (b) 2-electron Si etched widths vs reaction time.

This is further corroborated by Transmission Electron Microscopy (TEM), which reports SiNP diameters of ≈ 120 nm (Figure 3c), mostly porous, embedding small amounts of nanocrystals (Figure 3d).

To confirm and clarify the process, we investigated the sample by means of Raman spectroscopy to probe locally the structural properties of the material. In Figure 5a we present four representative Raman spectra, collected using a 532 nm excitation, on a 4.5 μm long SiNP, which was cleaved after the treatment and observed in cross-section. The optical investigation depth of the laser of about 0.4 μm^34^ can exclude artifacts related to the cleavage procedure. We repeated the experiment on different samples, which all showed similar results. The spectra were collected by scanning from bulk (crystalline silicon) toward SiNP tips. Three main regions were found. The spectrum from the deeper region with bulk crystalline Si, marked with sc-Si in the figure, displays a strong Fano resonance with an asymmetric band leaning toward higher wavenumbers, with a maximum at 516.7 cm^–1^.^35^ This resonance was expected because of the high doping level, which is also confirmed by the presence of the two satellite peaks due to vibration of the acceptors themselves (^10^B and ^11^B, 618 and 640 cm^–1^, respectively).^36,37^ Moving into the intermediate porous subsurface region, marked with por-Si in Figure 5a, the main crystalline band is still asymmetrical toward higher wavenumbers. This feature is consistent with a macroporous Si layer, with relatively large crystals preserving electronic level delocalization and Fano resonance thereof, in agreement with Li’s report.^13^ The two boron-related bands begin to merge forming a single broad feature centered at about 631 cm^–1^. In addition, we observe the appearance of a band at 480 cm^–1^, which is typical of disordered silicon.^38–40^ Finally, the spectra from the third region, marked with SiNP in Figure 5a, show an asymmetric band leaning toward lower wavenumbers, both on the base and at the tip of the wires. This asymmetry is a typical fingerprint of a significant reduction of the size of the silicon crystals, which is detectable from the relaxation of the Raman selection rule of the center of the Brillouin zone.^41^ For comparison, in Si quantum dots the asymmetry becomes measurable below about 10 nm.^42^ At the same time, the high wavenumber Fano resonance observed in the bulk disappears. Both these results suggest that the more extended exposure to the etching solution turned macroporous Si into nanoporous, with the localization of Si electronic states. Consistently, pore size is now too small to allow HF to diffuse in, so pore surfaces remain hydrophilic (oxidized) even upon extended exposure to HF, well explaining the observed irreversible SiNP bundling. Further confirmation of such macro-to-nanoporous conversion comes from the evolution of the spectra from the porous sublayer toward the tip of the wires. We collected a map of Raman spectra with a step size of 0.2 μm. Figure 5b shows the comparative analysis of the full width at half-maximum (FWHM) of the crystalline and disordered silicon bands. While a constant and small FWHM is observed in the porous layer region, located between −5 and −4 μm below the SiNP tips, the width dramatically increases moving from the base to the tip of the nanopillars, roughly from −4 to −2 μm, reaching a nearly stable value in the top 2 μm.

**Figure 5:**
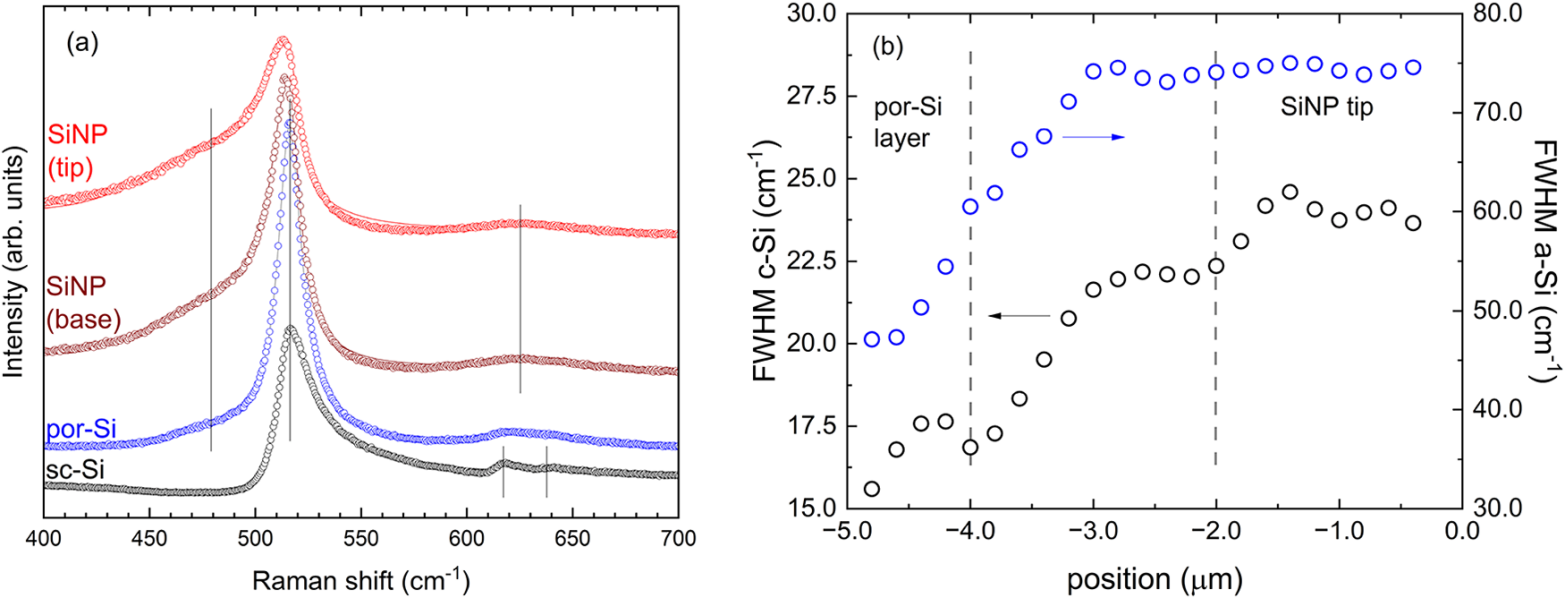
(a) Comparison of Raman spectra of the bulk (crystalline) silicon, the porous silicon layer, and the SiNPs. (b) Increase of the FWHM of the resonance at 480 cm^–1^ across the SiNPs, moving from their base to the tip.

In summary, Raman spectra confirm that the MACE process begins with the formation of macroporous Si layer, on which metal-assisted localized etch takes place. While MACE occurs at a higher rate than noncatalyzed etch, depth of porosization is self-limited (cf. Figure 4), in accordance with the model we advanced. As the process continues, SiNPs remain exposed to the etching solution, so that SiNPs evolve from macro to nanoporous, as witnessed by Raman analyses.

## Conclusions

We showed how a stronger oxidizing agent such as sodium peroxydisulfate, with a redox potential of 2.01 V vs SHE, as opposed to hydrogen peroxide (redox potential of 1.77 V vs SHE), leads to a reduction of carrier density at the Si–solution bare interface, decreasing the competitive noncatalyzed etching rate. This resulted in the unprecedented formation of porous SiNPs by MACE on degenerate *p*–type silicon with a boron density of 10^20^ cm^–3^. For several applications, Si porosity is either irrelevant (e.g., when SiNPs are used as antireflective layers) or even an additional advantage (e.g., for sensors and biosensors), where the extended surface area enables higher sensitivity.^43^ Instead, porosity is an undesirable feature for thermoelectric applications, as it decreases Si electrical conductivity more than its thermal conductivity. However, it has been shown^44^ that porous SiNPs of comparable doping level, obtained by nanoimprint lithography (NIL), display anyway a thermoelectric figure of merit *zT* > 0.5 at 500 K. Similar conclusions were achieved more recently by other scholars.^19^ Thus, comparing nanoporous SiNPs with a doping level of 10^20^ cm^–1^ with crystalline SiNPs with a doping level of ≈ 10^18^ cm^–1^, namely the highest doping level at which 4-electron etching prevails over 2-electron etching in MACE,^33^ it is apparent that the reduction of carrier mobility due to porosity remains largely overcompensated by the increase of carrier density. The concurrent reduction of thermal conductivity brings then to an enhanced figure of merit when SiNPs are manufactured by MACE. Although larger *zT* values can be achieved in single-crystalline Si nanowires by using extreme lithography,^2^ competitiveness and economic profitability of thermoelectric generators depend on the levelized cost of energy they provide, not just on their efficiency. Thus, considering the simplicity of the MACE approach (also compared to NIL) and the geo-abundance of silicon, our results are a remarkable leap toward more extensive applications of thermoelectricity to recover waste heat. Future work on MACE leading to degenerate SiNPs will address etching rate and its dependence on temperature, with the aim of extending to the 10^20^ cm^–1^ doping range the possibility of fully etching away the sustaining residual silicon membrane, as reported in low-doping SiNPs,^32^ enabling the making of self-sustaining SiNP pads.

## Experimental Section

### Materials Preparation

MACE on heavily doped *p*-type Si was carried out using a discontinuous, nanoporous Au layer as the catalyst and Na_2_S_2_O_8_ as the oxidizing agent. Degenerate *p*-type (100) Si wafers, boron-doped with a resistivity of 0.001 Ω cm (boron density: 1.0 × 10^20^ cm^–3^) were used. After cleansing with an APM (Ammonium Peroxide Mixture) solution (NH_3_ (29% vol.):H_2_O_2_ (33% vol.): H_2_O, 1:1:5) for 15 min at 65 °C,^45^ a 12 nm layer of gold was deposited by electron-beam evaporation. Since Au adhesion on Si is known to be poor, prior to gold deposition a 3 nm thick Ti layer was evaporated. Samples were soaked in deionized water kept at 20 °C in a thermostatic bath and then immersed in a solution containing Na_2_S_2_O_8_ (0.04 M) and HF (5 M) that was kept at 20 °C for the whole MACE duration, still using a thermostatic bath. Etching lasted from 15 to 350 min keeping the sample under agitation.

### Morphological Analyses

SEM images were collected using a ThermoFisher Phenom G6 SEM, equipped with a thermionic emission source (nominal resolution <6 nm at 15 kV) using a Everhart–Thornley type detector for secondary electrons and a standard solid state backscattered electron detector. SEM confirmed the discontinuous morphology of the Au layer. TEM micrographs were acquired using a JEOL JEM-2100 Plus Microscope equipped with a LaB_6_ electron source and operating at an accelerating voltage ranging from 80 to 200 kV. The nominal spatial resolution is 0.24 nm. Image acquisition was performed using a Gatan camera with an 8-megapixel sensor.

### Raman Analyses

Raman measurements were performed with a Jobin-Yvon T64000 spectrometer with an excitation wavelength of 532 nm and an excitation power of about 4 mW. The spectra were collected with a 100 × 0.90NA objective. Maps were collected in 0.2-μm steps.

## †

Department of Environmental Sciences, Informatics and Statistics, Ca’ Foscari University of Venice, via Torino 155, I–30170 Venezia Mestre, Italy

## References

[1] Hochbaum A. I., Chen R. K., Delgado R. D., Liang W. J., Garnett E. C., Najarian M., Majumdar A., Yang P. D. (2008). Enhanced thermoelectric
performance of rough silicon nanowires. Nature.

[2] Boukai A. I., Bunimovich Y., Tahir-Kheli J., Yu J., Goddard W., Heath J. (2008). Silicon nanowires
as efficient thermoelectric materials. Nature.

[3] Peng K., Xu Y., Wu Y., Yan Y., Lee S.-T., Zhu J. (2005). Aligned single-crystalline
Si nanowire arrays for photovoltaic applications. Small.

[4] Pera D. M., Costa I., Serra F., Gaspar G., Lobato K., Serra J. M., Silva J. A. (2023). Development of a metal-assisted chemical
etching method to improve light-capture in monocrystalline silicon
solar cells. Sol. Energy Mater. Sol. Cells.

[5] Chan C. K., Peng H., Liu G., McIlwrath K., Zhang X., Huggins R., Cui Y. (2008). High performance
lithium
battery anodes using silicon nanowires. Nat.
Nanotechnol..

[6] Mateen A., Khan A. J., Zhou Z., Mujear A., Farid G., Yan W., Li H., Li J., Bao Z. (2025). Silicon Nanowires via
Metal-Assisted Chemical Etching for Energy Storage Applications. ChemSusChem.

[7] Zhou X., Hu J., Li C., Ma D., Lee C., Lee S. (2003). Silicon nanowires
as chemical sensors. Chem. Phys. Lett..

[8] Zheng G., Patolsky F., Cui Y., Wang W. U., Lieber C. M. (2005). Multiplexed
electrical detection of cancer markers with nanowire sensor arrays. Nat. Biotechnol..

[9] Snyder G. J., Toberer E. S. (2008). Complex thermoelectric materials. Nat. Mater..

[10] Narducci D., Giulio F. (2022). Recent Advances on
Thermoelectric Silicon for Low-Temperature
Applications. Materials.

[11] Li X. (2012). Metal assisted
chemical etching for high aspect ratio nanostructures: A review of
characteristics and applications in photovoltaics. Curr. Opin. Solid State Mater. Sci..

[12] Huang Z., Geyer N., Werner P., De Boor J., Gösele U. (2011). Metal-assisted
chemical etching of silicon: a review: in memory of Prof. Ulrich Gösele. Adv. Mater..

[13] Li X., Bonn P. (2000). Metal-assisted chemical etching in HF/H_2_O_2_ produces
porous silicon. Appl. Phys. Lett..

[14] Chartier C., Bastide S., Lévy-Clément C. (2008). Metal-assisted chemical
etching of silicon in HF-H2O2. Electrochim.
Acta.

[15] Zhang M.-L., Peng K.-Q., Fan X., Jie J.-S., Zhang R.-Q., Lee S.-T., Wong N.-B. (2008). Preparation of large-area uniform
silicon nanowires arrays through metal-assisted chemical etching. J. Phys. Chem. C.

[16] Janavicius L. L., Michaels J., Chan C., Sievers D., Li X. (2023). Programmable
vapor-phase metal-assisted chemical etching for versatile high-aspect
ratio silicon nanomanufacturing. Appl. Phys.
Rev..

[17] Gupta S., Mishra D., DasMahapatra S., Singh K. (2024). Integration of silicon
nanostructures for health and energy applications using MACE: a cost-effective
process. Nanotechnology.

[18] Shim B., Park K.-R., Kim H., Kim C., Song Y., Kim W.-B. (2025). Scalable fabrication of ultra-long
silicon nanowires
via H_2_O_2_-enhanced MACE for flexible hydrogen
sensors. Chem. Eng. J..

[19] Toan N. V., Li Y., Tuoi T. T. K., Sabran N. S., Kiat J. H., Voiculescu I., Ono T. (2025). Thermoelectric generator using nanoporous silicon formed by metal-assisted
chemical etching method. Energy Convers. Manage..

[20] Suh J., Lee J., Kim J., Cho M., Kim J., Oh J., Han H., Lee H., Oh J. (2025). High Aspect Ratio Silicon Nanohole
Arrays via Electric-Field-Incorporated Metal-Assisted Chemical Etching. ACS Appl. Mater. Interfaces.

[21] Leonardi A. A., Faro M. J. L., Irrera A. (2021). Silicon Nanowires
Synthesis by Metal-Assisted
Chemical Etching: A Review. Nanomaterials.

[22] Leonardi A. A., Arrigo A., Lo Faro M. J., Nastasi F., Irrera A. (2024). 2D Fractal
Arrays of Ultrathin Silicon Nanowires as Cost-Effective and High-Performance
Substrate for Supercapacitors. Adv. Energy Sustainability
Res..

[23] To W.-K., Tsang C.-H., Li H.-H., Huang Z. (2011). Fabrication
of n-type
mesoporous silicon nanowires by one-step etching. Nano Lett..

[24] Qi Y., Wang Z., Zhang M., Yang F., Wang X. (2013). A processing
window for fabricating heavily doped silicon nanowires by metal-assisted
chemical etching. J. Phys. Chem. C.

[25] Lai R. A., Hymel T. M., Narasimhan V. K., Cui Y. (2016). Schottky Barrier Catalysis
Mechanism in Metal-Assisted Chemical Etching of Silicon. ACS Appl. Mater. Interfaces.

[26] Li H., Kato S., Soga T. (2022). Etching rate
of silicon nanowires
with highly doped silicon during metal-assisted chemical etching. Mater. Res. Express.

[27] Smith Z. R., Smith R. L., Collins S. D. (2013). Mechanism of nanowire
formation in
metal assisted chemical etching. Electrochim.
Acta.

[28] Elyamny S., Dimaggio E., Magagna S., Narducci D., Pennelli G. (2020). High power
thermoelectric generator based on vertical silicon nanowires. Nano Lett..

[29] Giulio F., Mazzacua A., Calciati L., Narducci D. (2024). Fabrication
of Metal
Contacts on Silicon Nanopillars: The Role of Surface Termination and
Defectivity. Materials.

[30] Kondos P., Deschênes G., Morrison R. (1995). Process optimization studies in gold
cyanidation. Hydrometallurgy.

[31] Wang S., Wu J., Jiao F. (2025). Pretreatment
and Extraction of Gold from Refractory
Gold Ore in Acidic Conditions. Minerals.

[32] Giulio F., Puccio L., Magagna S., Perego A., Mazzacua A., Narducci D. (2024). Self-Sustained Quasi-1D
Silicon Nanostructures for
Thermoelectric Applications. ACS Appl. Electron.
Mater..

[33] Magagna S., Narducci D., Alfonso C., Dimaggio E., Pennelli G., Charaï A. (2020). On the mechanism ruling the morphology of silicon nanowires
obtained by one-pot metal-assisted chemical etching. Nanotechnology.

[34] Aspnes D. E., Studna A. A. (1983). Dielectric functions
and optical parameters of Si,
Ge, GaP, GaAs, GaSb, InP, InAs, and InSb from 1.5 to 6.0 eV. Phys. Rev. B.

[35] Magidson V., Beserman R. (2002). Fano-type interference in the Raman spectrum of photoexcited
Si. Phys. Rev. B.

[36] Chandrasekhar M., Chandrasekhar H. R., Grimsditch M., Cardona M. (1980). Study of the localized
vibrations of boron in heavily doped Si. Phys.
Rev. B.

[37] Stutzmann M. (1987). Hydrogen passivation
of boron acceptors in silicon: Raman studies. Phys. Rev. B.

[38] Bermejo D., Cardona M. (1979). Raman scattering in pure and hydrogenated amorphous
germanium and silicon. J. Non-Cryst. Solids.

[39] Maslova N. E., Antonovsky A. A., Zhigunov D. M., Timoshenko V. Y., Glebov V. N., Seminogov V. N. (2010). Raman studies
of silicon nanocrystals
embedded in silicon suboxide layers. Semiconductors.

[40] Gaisler S. V., Semenova O. I., Sharafutdinov R. G., Kolesov B. A. (2004). Analysis of Raman
spectra of amorphous-nanocrystalline silicon films. Phys. Solid State.

[41] Campbell I. H., Fauchet P. M. (1986). The effects of microcrystal size and shape on the one
phonon Raman spectra of crystalline semiconductors. Solid State Commun..

[42] Faraci G., Gibilisco S., Russo P., Pennisi A. R., La Rosa S. (2006). Modified Raman
confinement model for Si nanocrystals. Phys.
Rev. B.

[43] Qin Y., Jiang Y., Zhao L. (2018). Modulation
of agglomeration of vertical
porous silicon nanowires and the effect on gas-sensing response. Adv. Eng. Mater..

[44] Yang L., Huh D., Ning R., Rapp V., Zeng Y., Liu Y., Ju S., Tao Y., Jiang Y., Beak J. (2021). High thermoelectric
figure of merit of porous Si nanowires from 300 to 700 K. Nat. Commun..

[45] Hull, R. Properties of crystalline silicon; INSPEC, The Institution of Electrical Engineers, 1999; p 1042.

